# History of a Case of Long Standing Spinal Irritation, Successfully Treated

**Published:** 1841-11

**Authors:** John Dawson

**Affiliations:** Jamestown, Ohio


Art. IV.—*History of a Case of long standing Spinal Irritation, successfully treated.* By John Dawson, Jr., of Jamestown, Ohio.

Since Teale published his work referring all nervous diseases to irritation of the nervous centres and ganglia of the sympathetic nerve, the profession has had its attention turned towards these localities as very prolific sources of disease. Charmed with the claims of the work, and the light it shed upon a class of diseases imperfectly understood, we naturally ran off on the hobby. Now, however, the medical mind is about becoming balanced again; and although we find a great majority of nervous affections generated and sustained by irritation of the nervous centres, others, it must be confessed, are supported by no such pathological condition.

Conceiving that well marked cases of spinal and ganglionic irritation are entitled to a passing notice, I give you a few notes on one of several which came under my observation.

The case referred to is that of Mr. J. F., 45 years of age. The history he gave me of his case was, that about 8 years ago he suffered from pain in the lumbar region; had also indigestion. These continued with intervals up to September, 1839, at which time I was called to prescribe.

*Symptoms.*—Many of these were anomalous and cannot be described. The patient would frequently say that his feelings were so miserable and so singular that he could not describe them. He was, nevertheless, a very intelligent farmer. Obvious enough, however, were others. Deep-seated pain in the spinal column, increased by pressure. This developed itself first in the lumbar region, then in the back, and lastly in the neck. Whilst confined to the lumbar region he was afflicted with drawing tingling sensations along the course of the nerves of the inferior extremities, which could be increased at any time by pressure at the origin of these nerves. When the pain occupied the dorsal part of the column the pain was less violent. The patient then complained of difficult breathing, tightness of the epigastrium, and the sensation of a cord being tied around the waist. Occasionally he suffered from spasms and tremors of the arms, which, with the hands, he generally kept in a painfully flexed condition. The distress in the cervical region was violent, and it multiplied greatly the previous symptoms. The head now became the seat of "roaring wind" sensations, cephalalgia, fulness alternating with unnatural emptiness of the encephalon, and a sharp irritableness of the intellectual faculties.

Together with the proper cervical nerves he now evidently suffered from disease of the eighth pair and sympathetic ganglia. Evidence conclusive of this existed in the fact, that the involuntary muscles, the heart, diaphragm, stomach, intestines, and trunks of large arteries, became perverted in their functions, as must necessarily have been the case when the nerves appropriated to these organs become in any manner affected. The heart was so much disturbed in its functions that at one time I supposed it to be organically diseased. Irregular and spasmodic contractions were of every day occurrence. Equally distressed were the thoracic viscera. It seemed here to simulate the *dry asthma* of old writers, producing a sense of smothering and great pulmonary oppression. Seated in the stomach was a sense of distension alternating with emptiness and hunger, when the patient would be compelled to eat. Very often the stomach secreted quantities of a viscid transparent liquid constituting what is called pyrosis. Perverted function, both in the large and small intestines, tended to produce successively, constipation, diarrhœa, and the secretion of gaseous exhalations.

The above is an outline, and but an outline, of the symptoms presented in this case of disease.

*Treatment.*—This, from beginning to end, occupied a period of nearly eighteen months. When first called, the first passages were emptied, and recourse had to anti-spasmodics, and medicines calculated to improve the perverted condition of the secretions. Failing entirely to accomplish any thing, attention was now directed to the spine. This consisted successively of all the modes of counter-irritation. We commenced by cupping and blistering; tartar-emetic ointment was then used to produce three or four crops of pustules. These produced in their turn, a mitigation of suffering, but were, on account of inconvenience, abandoned for the use of seatons. Palliatives, anodynes, and anti-spasmodics, were used to allay emergent suffering throughout the whole course of the treatment.

Reflection on this case proves—

1st. That a great variety of symptoms may be produced by a single disease.

2nd. That irritation of the sympathetic nerve, co-operating with a similar affection of the spinal nerves, multiplies in an eminent degree the symptoms, and embarrasses the cure.

3rd. The great indication to be fulfilled, is counter-irritation over the seat of the disorder.

4th. The length of time required to cure is sometimes very great, this patient having been eighteen months under medical treatment.

*February*, 1841.

